# 2-(4-Chloro­phen­yl)-4-phenyl-1,2-di­hydro­quinazoline

**DOI:** 10.1107/S1600536813027839

**Published:** 2013-10-19

**Authors:** Chamseddine Derabli, Raouf Boulcina, Sofiane Bouacida, Hocine Merazig, Abdelmadjid Debache

**Affiliations:** a Laboratoire de Synthèse des Molécules d’Intérêts Biologiques, Département de Chimie, Faculté des Sciences Exactes, Université de Constantine 1, 25000 Constantine, Algeria; b Unité de Recherche de Chimie de l’Environnement et Moléculaire Structurale, CHEMS, Université Constantine 1, 25000 , Algeria; c Département Sciences de la Matière, Faculté des Sciences Exactes et Sciences de la Nature et de la Vie, Université Oum El Bouaghi 04000, Algeria

## Abstract

In the title compound, C_20_H_15_ClN_2_, the pyrimidine ring is in a flattened half-chair conformation. The phenyl and chloro-substituted benzene rings form dihedral angles of 84.97 (5) and 80.23 (4)°, respectively, with the benzene ring of the di­hydro­quinazoline group. The dihedral angle between the phenyl and chloro-substituted benzene rings is 61.71 (5)°. In the crystal, mol­ecules are arranged in inter­secting layers parallel to (101) and (-102), with N—H⋯N hydrogen bonds linking mol­ecules along [010]. In addition, a weak C—H⋯π inter­action is observed.

## Related literature

For the preparation and applications of quinazoline derivatives, see: Gundla *et al.* (2008 ▶); Luth & Lowe (2008 ▶); Fry *et al.* (1994 ▶); Kunes *et al.* (2000 ▶); Michael (2002 ▶); Frère *et al.* (2003 ▶); Langer & Bodtke (2003 ▶).
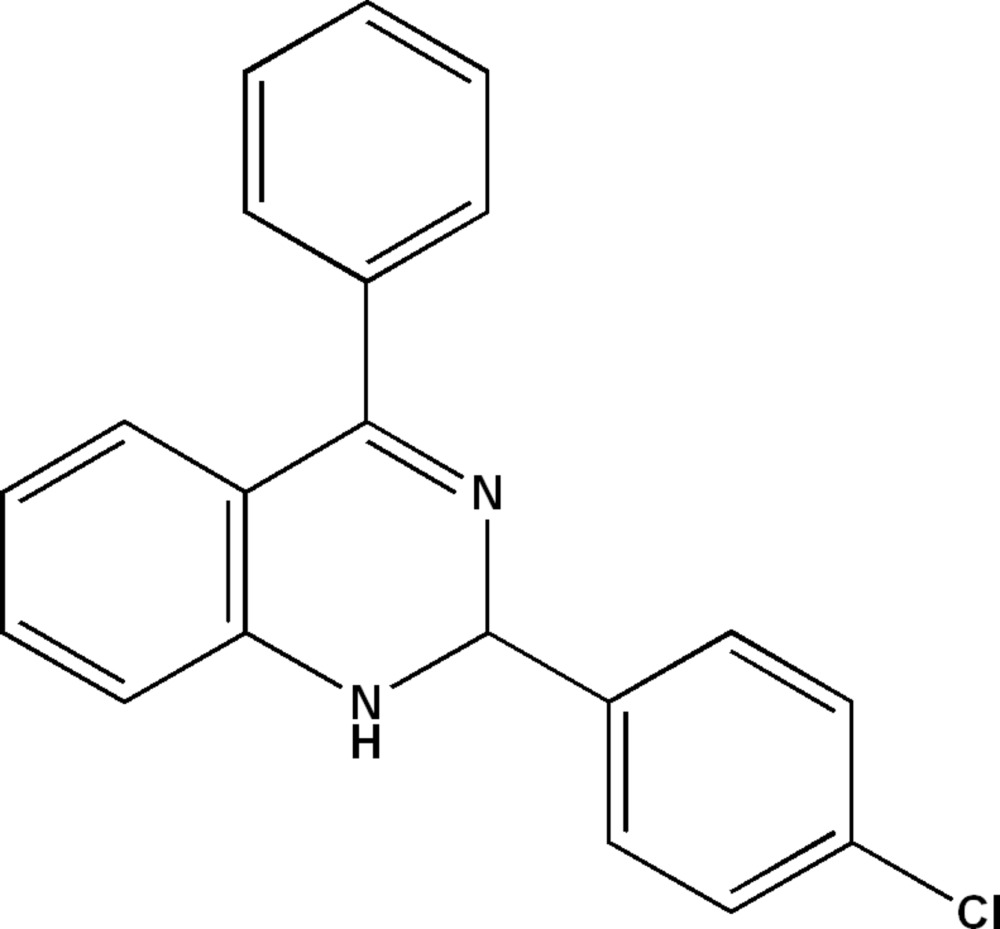


## Experimental

### 

#### Crystal data

C_20_H_15_ClN_2_
*M* *_r_* = 318.79
Monoclinic, 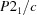

*a* = 9.2563 (10) Å
*b* = 10.6283 (11) Å
*c* = 17.6230 (19) Å
β = 116.775 (7)°
*V* = 1547.8 (3) Å^3^
*Z* = 4
Mo *K*α radiation
μ = 0.25 mm^−1^
*T* = 150 K
0.18 × 0.04 × 0.03 mm

#### Data collection

Bruker APEXII CCD area-detector diffractometer
Absorption correction: multi-scan (*SADABS*; Sheldrick, 2002 ▶) *T*_min_ = 0.948, *T*_max_ = 1.000
8914 measured reflections
2724 independent reflections
2462 reflections with *I* > 2σ(*I*)
*R*_int_ = 0.027

#### Refinement

*R*[*F* ^2^ > 2σ(*F* ^2^)] = 0.031
*wR*(*F* ^2^) = 0.081
*S* = 1.05
2724 reflections
212 parameters
H atoms treated by a mixture of independent and constrained refinement
Δρ_max_ = 0.22 e Å^−3^
Δρ_min_ = −0.26 e Å^−3^

### 

Data collection: *APEX2* (Bruker, 2011 ▶); cell refinement: *SAINT* (Bruker, 2011 ▶); data reduction: *SAINT*; program(s) used to solve structure: *SIR2002* (Burla *et al.*, 2003 ▶); program(s) used to refine structure: *SHELXL97* (Sheldrick, 2008 ▶); molecular graphics: *ORTEP-3 for Windows* (Farrugia, 2012 ▶) and *DIAMOND* (Brandenburg & Berndt, 2001 ▶); software used to prepare material for publication: *WinGX* (Farrugia, 2012 ▶).

## Supplementary Material

Crystal structure: contains datablock(s) I. DOI: 10.1107/S1600536813027839/lh5660sup1.cif

Structure factors: contains datablock(s) I. DOI: 10.1107/S1600536813027839/lh5660Isup2.hkl

Supplementary material file. DOI: 10.1107/S1600536813027839/lh5660Isup3.cml

Additional supplementary materials:  crystallographic information; 3D view; checkCIF report

## Figures and Tables

**Table 1:** Hydrogen-bond geometry (Å, °) *Cg* is the centroid of the C15–C20 ring.

*D*—H⋯*A*	*D*—H	H⋯*A*	*D*⋯*A*	*D*—H⋯*A*
N1—H1*N*⋯N2^i^	0.830 (19)	2.316 (19)	3.1234 (18)	164.3 (19)
C11—H11⋯*Cg* ^ii^	0.93	2.76	3.666 (2)	165

Symmetry codes: (i) 
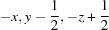
; (ii) 
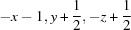
.

**Fig. 1.**
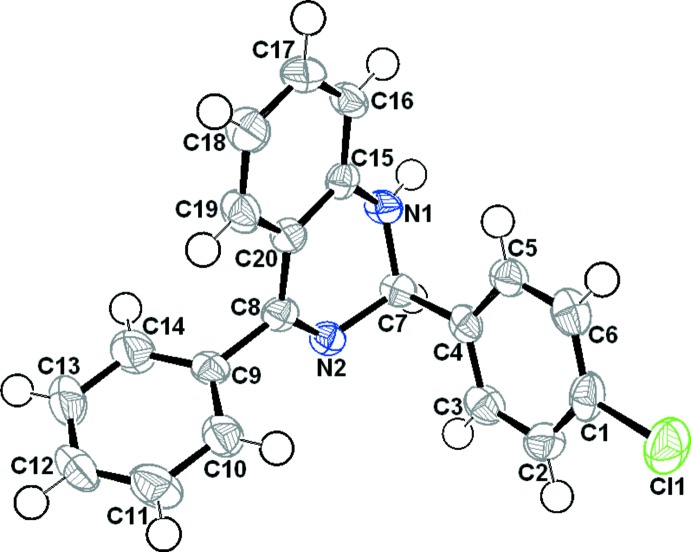
The molecular structure with displacement ellipsoids drawn at the 50% probability level.

**Fig. 2.**
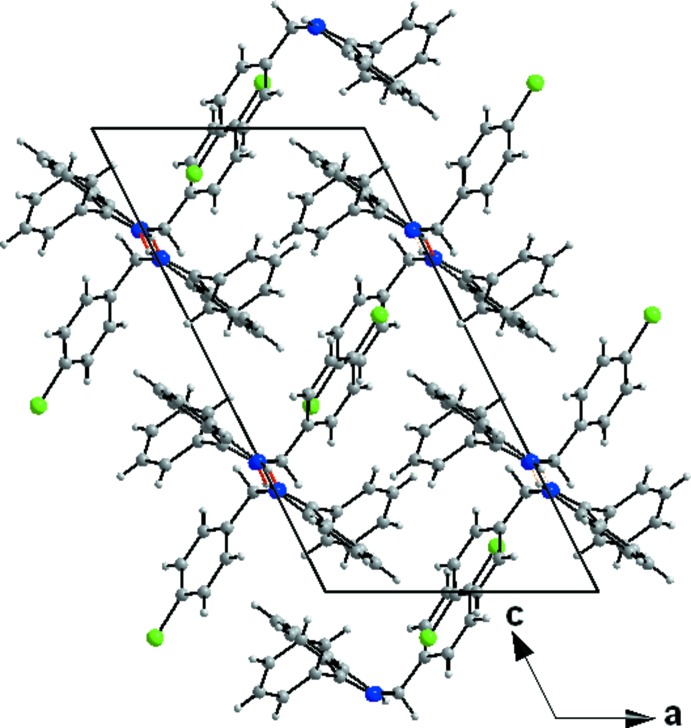
Part of the crystal structure viewed along the b axis. Cl is shown in green, N in blue and C in grey. Red lines indicate hydrogen bonds.

**Fig. 3.**
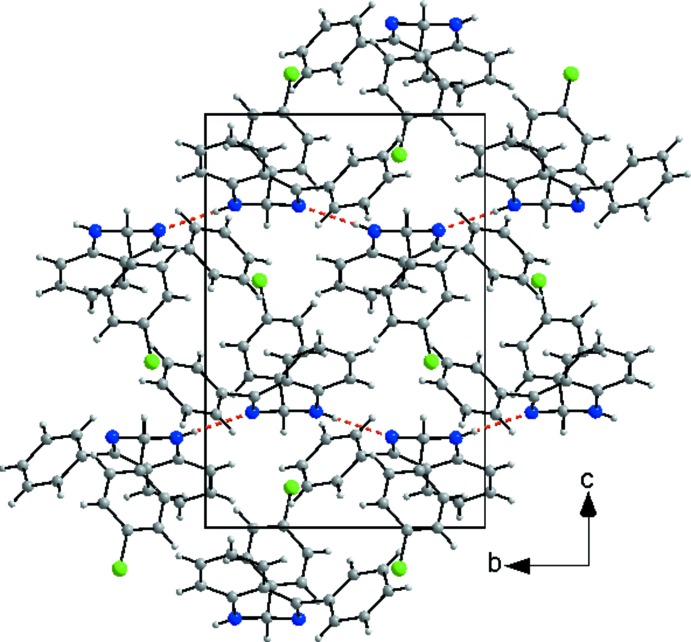
Part of the crystal structure showing the hydrogen bonds N—H···N as dashed red lines.

## supplementary crystallographic information

### 1. Comment

Heterocyclic chemistry is a potential part of the synthetic organic chemistry, covering a wide variety of bioactive molecules. Among six-membered heterocycles, quinazoline occupies significant position and is commonly found in a wide variety of natural products, synthetic pharmaceutical molecules, and other functional materials (Gundla *et al.*, 2008; Luth & Lowe, 2008). Quinazoline derivatives are among the most potent tyrosine kinase and cellular phosphorylation inhibitors (Fry *et al.*, 1994), and they also show remarkable activity as antitubercular, antiviral, and anticancer agents (Kunes *et al.*, 2000). The growing medicinal importance of these heterocycles perpetuates to provide strong rationale for the development of synthetic methods for their preparation. These efforts have led to several reviews emphasizing the synthesis (Michael, 2002; Frère *et al.*, 2003; Langer & Bodtke, 2003), and biological evaluation of quinazolines.

In the course of a program directed toward the synthesis of new heterocyclic systems for pharmacological evaluation, we report herein the crystallographic study and the synthesis of the title compound. The molecular structure is shown in Fig. 1. The phenyl ring and chloro-substituted benzene rings form a dihedral angles of 84.97 (5) and 80.23 (4)° respectively with the benzene ring of dihydroquinazoline group. The dihedral angle between the phenyl ring and chloro-substituted benzene ring is 61.71 (5) °. In the crystal, molecules are arranged in intersecting layers parallel to (101) and (-102) (see, Fig. 2) with N—H···N hydrogen bonds linking molecules along [010] (Fig. 3). In addition, a weak C—H···π interaction is observed (Table 1).

### 2. Experimental

The title compound was prepared by condensation of 4-chlorobenzaldehyde (1.0 equiv), 2-aminobenzophenone (1.0 equiv), ammonium acetate (2.0 equiv), and dimethylaminopyridine (0.2 equiv.) in 5 ml of absolute ethanol at 313 K. After completion of the reaction as monitored by TLC, the reaction was poured into ice cold water; solid product was filtered, washed with water and dried. The crude product was recrystallized from ethyl acetate to give the tite compound as a yellow solid (m.p. 415–417 K). X-ray quality crystals were grown from a solution of the title compound in ethyl acetate.

### 3. Refinement

H atoms bonded to C atoms were initially located in a difference Fourier map. However, they were subsequently placed in idealized positions and refined in a riding-model approximation. The applied constraints were as follows: C_aryl_—H_aryl_ = 0.93 Å; C_methine_—H_methine_ = 0.98 Å; *U*_iso_(H_aryl_H_methine_) = 1.2*U*_eq_(C_aryl_/C_methine_). Atom H1N was located in a difference Fourier map and refined isotropically.

### Crystal data

**Table:**

C_20_H_15_ClN_2_	*F*(000) = 664
*M**_r_* = 318.79	*D*_x_ = 1.368 Mg m^−^^3^
Monoclinic, *P*2_1_/*c*	Mo *K*α radiation, λ = 0.71073 Å
Hall symbol: -P 2ybc	Cell parameters from 4726 reflections
*a* = 9.2563 (10) Å	θ = 2.5–25.1°
*b* = 10.6283 (11) Å	µ = 0.25 mm^−^^1^
*c* = 17.6230 (19) Å	*T* = 150 K
β = 116.775 (7)°	Needle, colourless
*V* = 1547.8 (3) Å^3^	0.18 × 0.04 × 0.03 mm
*Z* = 4	

### Data collection

**Table:**

Bruker APEXII CCD area-detector diffractometer	2462 reflections with *I* > 2σ(*I*)
Graphite monochromator	*R*_int_ = 0.027
φ and ω scans	θ_max_ = 25.1°, θ_min_ = 2.3°
Absorption correction: multi-scan (*SADABS*; Sheldrick, 2002)	*h* = −10→11
*T*_min_ = 0.948, *T*_max_ = 1.000	*k* = −12→12
8914 measured reflections	*l* = −17→21
2724 independent reflections	

### Refinement

**Table:**

Refinement on *F*^2^	Primary atom site location: structure-invariant direct methods
Least-squares matrix: full	Secondary atom site location: difference Fourier map
*R*[*F*^2^ > 2σ(*F*^2^)] = 0.031	Hydrogen site location: inferred from neighbouring sites
*wR*(*F*^2^) = 0.081	H atoms treated by a mixture of independent and constrained refinement
*S* = 1.05	*w* = 1/[σ^2^(*F*_o_^2^) + (0.033*P*)^2^ + 0.9522*P*] where *P* = (*F*_o_^2^ + 2*F*_c_^2^)/3
2724 reflections	(Δ/σ)_max_ = 0.001
212 parameters	Δρ_max_ = 0.22 e Å^−^^3^
0 restraints	Δρ_min_ = −0.26 e Å^−^^3^

### Special details

**Table:**

Experimental. Spectroscopic data: IR (KBr) ν 3320, 2364 1620, 1537, 1486, 1321, 1263, 1155, 1015, 964, 805, 741, 697 cm-1; 1H NMR (CDCl3, 400 MHz) δ 7.72–7.61 (m, 5H, arom.), 7.49–7.36 (m, 4H, arom.), 7.32–7.21 (m, 2H, arom.), 6.77 (td, J=8.0,1.0 Hz, 1H, arom.), 6.72 (d, J=8.0 Hz, 1H), 6.02 (s, 1H, CH), 4.38 (s, 1H, NH); 13 C NMR (CDCl3, 100 MHz) δ 165.8, 146.9, 141.5, 141.4, 140.9, 138.1, 132.9, 130.2, 129.4, 129.3, 129.1, 128.1, 127.8, 127.5, 127.3, 127.2, 118.3, 117.9, 114.3, 72.4. Anal. calcd for C20H15N2Cl: C, 75.35; H, 4.74; N, 8.79; Found: C, 75.75; H, 4.95; N, 9.42. HRMS calcd for C_20_H_16_N_2_Cl (MH+) 319.0924; found 319.0863.
Geometry. All e.s.d.'s (except the e.s.d. in the dihedral angle between two l.s. planes) are estimated using the full covariance matrix. The cell e.s.d.'s are taken into account individually in the estimation of e.s.d.'s in distances, angles and torsion angles; correlations between e.s.d.'s in cell parameters are only used when they are defined by crystal symmetry. An approximate (isotropic) treatment of cell e.s.d.'s is used for estimating e.s.d.'s involving l.s. planes.
Refinement. Refinement of *F*^2^ against ALL reflections. The weighted *R*-factor *wR* and goodness of fit *S* are based on *F*^2^, conventional *R*-factors *R* are based on *F*, with *F* set to zero for negative *F*^2^. The threshold expression of *F*^2^ > σ(*F*^2^) is used only for calculating *R*-factors(gt) *etc*. and is not relevant to the choice of reflections for refinement. *R*-factors based on *F*^2^ are statistically about twice as large as those based on *F*, and *R*- factors based on ALL data will be even larger.

### Fractional atomic coordinates and isotropic or equivalent isotropic displacement parameters (Å^2^)

**Table:**

	*x*	*y*	*z*	*U*_iso_*/*U*_eq_	
C1	0.53121 (17)	0.77886 (14)	0.50548 (10)	0.0165 (3)	
C2	0.44580 (18)	0.66972 (14)	0.49960 (9)	0.0168 (3)	
H2	0.4841	0.6114	0.5437	0.02*	
C3	0.30186 (17)	0.64835 (14)	0.42695 (10)	0.0153 (3)	
H3	0.244	0.5748	0.4223	0.018*	
C4	0.24323 (17)	0.73546 (13)	0.36111 (9)	0.0130 (3)	
C5	0.33457 (18)	0.84305 (14)	0.36800 (10)	0.0161 (3)	
H5	0.2981	0.9009	0.3237	0.019*	
C6	0.47870 (18)	0.86503 (14)	0.43980 (10)	0.0177 (3)	
H6	0.5393	0.9367	0.4438	0.021*	
C7	0.07957 (17)	0.71954 (13)	0.28323 (9)	0.0131 (3)	
H7	0.0982	0.72	0.2327	0.016*	
C8	−0.10527 (16)	0.82529 (13)	0.31836 (9)	0.0124 (3)	
C9	−0.18848 (17)	0.94230 (13)	0.32554 (9)	0.0129 (3)	
C10	−0.35237 (19)	0.96183 (15)	0.27479 (11)	0.0239 (4)	
H10	−0.4123	0.903	0.2334	0.029*	
C11	−0.42727 (19)	1.06843 (15)	0.28539 (11)	0.0258 (4)	
H11	−0.5367	1.0821	0.2503	0.031*	
C12	−0.33965 (19)	1.15455 (14)	0.34810 (10)	0.0208 (3)	
H12	−0.3906	1.2252	0.356	0.025*	
C13	−0.1764 (2)	1.13562 (15)	0.39897 (10)	0.0228 (4)	
H13	−0.1174	1.1936	0.4412	0.027*	
C14	−0.10031 (18)	1.03001 (14)	0.38718 (10)	0.0188 (3)	
H14	0.0101	1.0182	0.4208	0.023*	
C15	−0.08004 (16)	0.59670 (13)	0.33333 (9)	0.0119 (3)	
C16	−0.12929 (16)	0.70970 (13)	0.35624 (9)	0.0129 (3)	
C17	−0.20595 (17)	0.70602 (14)	0.40861 (9)	0.0160 (3)	
H17	−0.2382	0.7806	0.424	0.019*	
C18	−0.23427 (18)	0.59305 (14)	0.43764 (10)	0.0180 (3)	
H18	−0.2842	0.5913	0.4731	0.022*	
C19	−0.18758 (18)	0.48132 (14)	0.41355 (10)	0.0171 (3)	
H19	−0.2075	0.405	0.4329	0.021*	
C20	−0.11248 (17)	0.48210 (13)	0.36158 (9)	0.0140 (3)	
H20	−0.0834	0.4068	0.3453	0.017*	
N1	−0.00529 (14)	0.60480 (12)	0.28194 (8)	0.0135 (3)	
N2	−0.01662 (14)	0.83324 (11)	0.27983 (7)	0.0129 (3)	
Cl1	0.70928 (4)	0.80771 (4)	0.59784 (2)	0.02398 (13)	
H1N	0.018 (2)	0.5377 (18)	0.2660 (11)	0.023 (5)*	

### Atomic displacement parameters (Å^2^)

**Table:**

	*U*^11^	*U*^22^	*U*^33^	*U*^12^	*U*^13^	*U*^23^
C1	0.0120 (7)	0.0221 (8)	0.0148 (7)	0.0023 (6)	0.0053 (6)	−0.0041 (6)
C2	0.0180 (7)	0.0189 (8)	0.0154 (7)	0.0047 (6)	0.0093 (6)	0.0039 (6)
C3	0.0161 (7)	0.0135 (7)	0.0193 (8)	0.0010 (6)	0.0107 (6)	0.0008 (6)
C4	0.0143 (7)	0.0136 (7)	0.0146 (7)	0.0027 (5)	0.0095 (6)	−0.0015 (5)
C5	0.0191 (8)	0.0151 (7)	0.0161 (7)	0.0019 (6)	0.0095 (6)	0.0024 (6)
C6	0.0168 (8)	0.0150 (7)	0.0221 (8)	−0.0014 (6)	0.0096 (6)	−0.0028 (6)
C7	0.0158 (7)	0.0122 (7)	0.0137 (7)	0.0001 (5)	0.0088 (6)	0.0001 (5)
C8	0.0122 (7)	0.0127 (7)	0.0098 (7)	−0.0014 (5)	0.0027 (6)	−0.0017 (5)
C9	0.0166 (7)	0.0106 (7)	0.0137 (7)	0.0001 (5)	0.0090 (6)	0.0029 (5)
C10	0.0194 (8)	0.0181 (8)	0.0263 (9)	0.0000 (6)	0.0034 (7)	−0.0073 (7)
C11	0.0157 (8)	0.0223 (8)	0.0329 (9)	0.0061 (6)	0.0050 (7)	0.0001 (7)
C12	0.0269 (9)	0.0144 (7)	0.0254 (9)	0.0071 (6)	0.0156 (7)	0.0023 (6)
C13	0.0283 (9)	0.0155 (8)	0.0203 (8)	0.0015 (6)	0.0073 (7)	−0.0045 (6)
C14	0.0169 (8)	0.0157 (8)	0.0197 (8)	0.0022 (6)	0.0047 (6)	−0.0002 (6)
C15	0.0101 (7)	0.0142 (7)	0.0097 (7)	−0.0006 (5)	0.0029 (5)	−0.0016 (5)
C16	0.0124 (7)	0.0125 (7)	0.0131 (7)	−0.0002 (5)	0.0051 (6)	−0.0005 (5)
C17	0.0184 (8)	0.0143 (7)	0.0184 (8)	0.0031 (6)	0.0109 (6)	−0.0007 (6)
C18	0.0217 (8)	0.0176 (8)	0.0219 (8)	0.0020 (6)	0.0162 (7)	0.0021 (6)
C19	0.0192 (8)	0.0129 (7)	0.0210 (8)	−0.0008 (6)	0.0105 (6)	0.0034 (6)
C20	0.0158 (7)	0.0100 (7)	0.0156 (7)	0.0012 (5)	0.0066 (6)	−0.0019 (5)
N1	0.0171 (6)	0.0115 (6)	0.0155 (6)	−0.0005 (5)	0.0104 (5)	−0.0031 (5)
N2	0.0139 (6)	0.0122 (6)	0.0118 (6)	−0.0003 (5)	0.0052 (5)	−0.0007 (5)
Cl1	0.0168 (2)	0.0295 (2)	0.0186 (2)	−0.00015 (15)	0.00177 (16)	−0.00224 (15)

### Geometric parameters (Å, º)

**Table:**

C1—C2	1.381 (2)	C10—H10	0.93
C1—C6	1.381 (2)	C11—C12	1.382 (2)
C1—Cl1	1.7443 (15)	C11—H11	0.93
C2—C3	1.389 (2)	C12—C13	1.381 (2)
C2—H2	0.93	C12—H12	0.93
C3—C4	1.389 (2)	C13—C14	1.390 (2)
C3—H3	0.93	C13—H13	0.93
C4—C5	1.395 (2)	C14—H14	0.93
C4—C7	1.528 (2)	C15—N1	1.3678 (19)
C5—C6	1.384 (2)	C15—C20	1.399 (2)
C5—H5	0.93	C15—C16	1.407 (2)
C6—H6	0.93	C16—C17	1.395 (2)
C7—N1	1.4452 (18)	C17—C18	1.376 (2)
C7—N2	1.4861 (18)	C17—H17	0.93
C7—H7	0.98	C18—C19	1.394 (2)
C8—N2	1.2820 (19)	C18—H18	0.93
C8—C16	1.462 (2)	C19—C20	1.377 (2)
C8—C9	1.4979 (19)	C19—H19	0.93
C9—C14	1.384 (2)	C20—H20	0.93
C9—C10	1.386 (2)	N1—H1N	0.831 (19)
C10—C11	1.384 (2)		
			
C2—C1—C6	121.37 (14)	C12—C11—H11	120
C2—C1—Cl1	119.08 (12)	C10—C11—H11	120
C6—C1—Cl1	119.56 (12)	C13—C12—C11	119.94 (14)
C1—C2—C3	119.04 (13)	C13—C12—H12	120
C1—C2—H2	120.5	C11—C12—H12	120
C3—C2—H2	120.5	C12—C13—C14	120.04 (15)
C2—C3—C4	120.82 (14)	C12—C13—H13	120
C2—C3—H3	119.6	C14—C13—H13	120
C4—C3—H3	119.6	C9—C14—C13	120.12 (14)
C3—C4—C5	118.71 (14)	C9—C14—H14	119.9
C3—C4—C7	122.18 (13)	C13—C14—H14	119.9
C5—C4—C7	119.07 (13)	N1—C15—C20	122.97 (13)
C6—C5—C4	121.00 (14)	N1—C15—C16	117.54 (12)
C6—C5—H5	119.5	C20—C15—C16	119.47 (13)
C4—C5—H5	119.5	C17—C16—C15	119.54 (13)
C1—C6—C5	119.00 (14)	C17—C16—C8	123.56 (13)
C1—C6—H6	120.5	C15—C16—C8	116.78 (13)
C5—C6—H6	120.5	C18—C17—C16	120.62 (13)
N1—C7—N2	111.97 (11)	C18—C17—H17	119.7
N1—C7—C4	114.81 (12)	C16—C17—H17	119.7
N2—C7—C4	106.24 (11)	C17—C18—C19	119.54 (14)
N1—C7—H7	107.9	C17—C18—H18	120.2
N2—C7—H7	107.9	C19—C18—H18	120.2
C4—C7—H7	107.9	C20—C19—C18	121.08 (13)
N2—C8—C16	124.23 (13)	C20—C19—H19	119.5
N2—C8—C9	117.79 (12)	C18—C19—H19	119.5
C16—C8—C9	117.99 (12)	C19—C20—C15	119.72 (13)
C14—C9—C10	119.50 (14)	C19—C20—H20	120.1
C14—C9—C8	118.79 (13)	C15—C20—H20	120.1
C10—C9—C8	121.65 (13)	C15—N1—C7	118.43 (12)
C11—C10—C9	120.30 (14)	C15—N1—H1N	117.2 (13)
C11—C10—H10	119.8	C7—N1—H1N	120.4 (13)
C9—C10—H10	119.8	C8—N2—C7	116.09 (12)
C12—C11—C10	120.06 (15)		
			
C6—C1—C2—C3	−1.8 (2)	C12—C13—C14—C9	−1.2 (2)
Cl1—C1—C2—C3	178.36 (11)	N1—C15—C16—C17	−179.97 (12)
C1—C2—C3—C4	−0.5 (2)	C20—C15—C16—C17	1.7 (2)
C2—C3—C4—C5	2.2 (2)	N1—C15—C16—C8	3.85 (19)
C2—C3—C4—C7	−175.49 (13)	C20—C15—C16—C8	−174.52 (12)
C3—C4—C5—C6	−1.8 (2)	N2—C8—C16—C17	170.14 (14)
C7—C4—C5—C6	176.00 (13)	C9—C8—C16—C17	−9.4 (2)
C2—C1—C6—C5	2.2 (2)	N2—C8—C16—C15	−13.8 (2)
Cl1—C1—C6—C5	−177.93 (11)	C9—C8—C16—C15	166.65 (12)
C4—C5—C6—C1	−0.4 (2)	C15—C16—C17—C18	−0.3 (2)
C3—C4—C7—N1	−2.54 (19)	C8—C16—C17—C18	175.64 (14)
C5—C4—C7—N1	179.78 (12)	C16—C17—C18—C19	−0.8 (2)
C3—C4—C7—N2	121.79 (14)	C17—C18—C19—C20	0.4 (2)
C5—C4—C7—N2	−55.89 (16)	C18—C19—C20—C15	1.0 (2)
N2—C8—C9—C14	−79.22 (17)	N1—C15—C20—C19	179.72 (13)
C16—C8—C9—C14	100.32 (16)	C16—C15—C20—C19	−2.0 (2)
N2—C8—C9—C10	103.78 (17)	C20—C15—N1—C7	−155.71 (13)
C16—C8—C9—C10	−76.68 (18)	C16—C15—N1—C7	25.99 (18)
C14—C9—C10—C11	0.3 (2)	N2—C7—N1—C15	−45.39 (17)
C8—C9—C10—C11	177.24 (15)	C4—C7—N1—C15	75.86 (16)
C9—C10—C11—C12	−1.6 (3)	C16—C8—N2—C7	−6.74 (19)
C10—C11—C12—C13	1.5 (3)	C9—C8—N2—C7	172.77 (12)
C11—C12—C13—C14	−0.1 (2)	N1—C7—N2—C8	34.69 (16)
C10—C9—C14—C13	1.1 (2)	C4—C7—N2—C8	−91.38 (14)
C8—C9—C14—C13	−175.93 (14)		

### Hydrogen-bond geometry (Å, º)

Cg is the centroid of the C15–C20 ring.

**Table:**

*D*—H···*A*	*D*—H	H···*A*	*D*···*A*	*D*—H···*A*
N1—H1*N*···N2^i^	0.830 (19)	2.316 (19)	3.1234 (18)	164.3 (19)
C3—H3···N1	0.93	2.53	2.878 (2)	102
C11—H11···*Cg*^ii^	0.93	2.76	3.666 (2)	165

Symmetry codes: (i) −*x*, *y*−1/2, −*z*+1/2; (ii) −*x*−1, *y*+1/2, −*z*+1/2.
